# Camel Milk Extracellular Vesicles as Engineered Biogenic Particles: Thermosensitive Hydrogel Integration for Optimized Wound Delivery and Tissue Regeneration

**DOI:** 10.3390/pharmaceutics18080943

**Published:** 2026-07-30

**Authors:** Shiqi Li, Rili Ge, Hui Yang

**Affiliations:** 1 College of Basic Medical Science, Qinghai University, Xining 810016, China; 2 Research Center for High Altitude Medicine, Qinghai University, Xining 810001, China

**Keywords:** drug delivery systems, particle engineering, biogenic nanoparticles, extracellular vesicles, thermosensitive hydrogel, system optimization, wound healing, controlled release, camel milk

## Abstract

**Objective:** This study aimed to enhance wound healing by developing a delivery platform that combines camel milk-derived extracellular vesicles (CM-EVs) with a thermosensitive chitosan/Poloxamer 407 hydrogel (CM-EVs–Gel), addressing the challenges of instability, poor skin penetration, and burst release associated with EVs. **Methods:** CM-EVs were isolated and analyzed for size, markers, and protein content. A thermosensitive hydrogel was created and infused with CM-EVs. Its gelation, injectability, and release kinetics (using the Higuchi model) were tested. Safety was evaluated through ocular irritation and 28-day skin toxicity in rabbits. Wound healing effectiveness was tested in rats with full-thickness wounds, comparing CM-EVs–Gel, a blank hydrogel, and untreated controls. **Results:** CM-EVs had an average size of 108.5 nm and expressed CD63, CD81, and Alix. The hydrogel solidified at 37 °C within 10 min and followed the Higuchi model for diffusion-controlled release (*R*^2^ = 0.974), releasing 81.7% of EVs over 48 h without toxicity. In rats, CM-EVs–Gel achieved 76.31% wound closure by day 6 and 94.7% by day 15, outperforming blank hydrogel (48.77% and 82.1%) and untreated controls (43.14% and 72.3%) (*p* < 0.01). Histology showed improved re-epithelialization, collagen deposition, and angiogenesis. **Conclusions:** This study shows that integrating biogenic particle engineering with optimized hydrogel systems allows for controlled release, safety, and enhanced wound healing. Despite missing free EV controls, full rheological data, and mechanistic insights, it highlights comprehensive delivery strategies from particle design to system performance, aligning with the Special Issue’s focus.

## 1. Introduction

Wound healing poses a significant and escalating challenge in clinical medicine, affecting millions of patients worldwide, with prevalence rising sharply among the elderly and individuals with comorbidities such as diabetes and vascular disease [1,2]. In China, the average healing duration for problematic wounds can extend to several months, severely compromising patients’ quality of life [3]. The socioeconomic burden is immense, with global wound care expenditures exceeding tens of billions of dollars annually [4].

The pathophysiology of impaired wound healing involves a prolonged inflammatory phase, impaired cell migration, insufficient angiogenesis, and extracellular matrix degradation, collectively shifting the balance from healing toward tissue breakdown [5]. Current therapies—including recombinant growth factors (e.g., becaplermin), advanced dressings, and negative pressure wound therapy—show variable outcomes and have significant limitations [6,7]. Becaplermin requires daily application due to its half-life of <30 min and bioavailability < 5%, while costs remain prohibitive [6,8]. These challenges have spurred interest in delivery platforms that protect bioactive agents, sustain release, and enable deep tissue penetration.

Extracellular vesicles (EVs), particularly those from milk, have emerged as promising natural nanocarriers for tissue regeneration [9,10]. EVs are lipid-bilayer nanoparticles (30–1000 nm) that carry proteins, lipids, and nucleic acids (e.g., miR-31, miR-214, VEGF, TGF-β), influencing inflammation, proliferation, angiogenesis, and matrix deposition [11,12]. Among milk-derived EVs, camel milk EVs (CM-EVs) offer distinct advantages. Proteomic analyses show that CM-EVs contain ~47% more lactoferrin than bovine milk EVs—a glycoprotein with pro-angiogenic and immunomodulatory functions [13,14]. CM-EVs are also enriched in integrin α5β1, which enhances matrix adhesion and increases bioactive retention by ~2.7-fold compared to integrin-deficient EVs [15]. These features position CM-EVs as attractive candidates for wound healing.

However, direct application of CM-EVs to wounds faces two major obstacles. First, wound microenvironments contain high levels of proteases and reactive oxygen species that degrade EV components [16]. Second, the stratum corneum can restrict passive EV penetration into deeper tissues where regeneration is needed [17]. An engineered delivery system that protects CM-EVs from degradation while enabling controlled release and transdermal transport is therefore required.

Temperature-sensitive hydrogels offer an innovative solution. These systems undergo reversible sol-to-gel transition at physiological temperature, allowing for minimally invasive liquid application followed by rapid in situ gelation [18]. Poloxamer 407 (Pluronic F-127) is widely used for its biocompatibility and reversible gelation [19]. Chitosan, a natural cationic polysaccharide, enhances the system with mucoadhesive, protease-inhibitory, and hemostatic properties [20,21]. The combination creates a synergistic matrix that protects encapsulated EVs and enables temperature-responsive controlled release.

While numerous EV-loaded hydrogel systems have been documented for wound healing, the majority predominantly utilize EVs sourced from cultured cell lines, such as mesenchymal stem cells or adipose-derived stem cells [22,23,24]. In contrast, our research presents a fundamentally novel approach by utilizing EVs derived from camel milk—a natural, abundantly available, and cost-effective source that has not been previously investigated for EV–hydrogel-based wound therapy. Notably, camel milk EVs exhibit a distinct bioactive cargo profile, characterized by exceptionally high levels of lactoferrin and integrin α5β1. This differentiates them from EVs derived from mammalian cells and imparts inherent anti-inflammatory, antimicrobial, and pro-regenerative properties, which are particularly beneficial for wound healing applications [13,14,15]. Moreover, whereas conventional EV–hydrogel systems generally incorporate EVs into basic polymer matrices, our approach distinctively integrates cardiac mesenchymal extracellular vesicles (CM-EVs) with a chitosan/Poloxamer 407 composite hydrogel. This integration transcends the mere application of previously documented components; it constitutes a deliberately engineered synergistic platform. In this system, chitosan imparts mucoadhesive and protease-inhibitory properties, while Poloxamer 407 provides thermosensitive injectability. This dual-functional synergy represents a novel achievement not previously realized with milk-derived EVs [20,21].

In this study, we utilized particle engineering principles to isolate and characterize well-defined cardiomyocyte-derived extracellular vesicle (CM-EV) biogenic nanoparticles, subsequently optimizing the system through hydrogel encapsulation. We hypothesized that the integration of engineered CM-EV particles with a thermosensitive hydrogel system would synergistically enhance controlled delivery, safety, and wound healing efficacy. This research aligns directly with the Special Issue’s objective of advancing complementary delivery strategies, encompassing both particle design and system performance.

## 2. Materials and Methods

### 2.1. Materials

Raw camel milk was collected from healthy lactating camels (*Camelus bactrianus*) in smallholder pastoral areas of Haixi Prefecture, Qinghai Province, China (March–April 2024). Chitosan (medium molecular weight, 50 kDa, deacetylation degree ≥ 75%, product No. C3646) and Poloxamer 407 (product No. P2443) were purchased from Sigma-Aldrich (St. Louis, MO, USA). Phosphate-buffered saline (PBS, pH 7.4, Gibco, Catalog No. 10010023, Waltham, MA, USA) was obtained from Thermo Fisher Scientific. All other analytical-grade reagents were obtained from commercial suppliers. Primary antibodies against CD63 (ab134045), CD81 (ab109201), and Alix (ab125011) were purchased from Abcam (Cambridge, UK). The PKH67 Green Fluorescent Cell Linker Kit (MINI67) was obtained from Sigma-Aldrich (St. Louis, MO, USA).

### 2.2. Isolation of Camel Milk Extracellular Vesicles

CM-EVs were isolated from raw camel milk by differential ultracentrifugation according to established protocols [15]. Briefly, raw milk was centrifuged at 300× *g* for 10 min at 4 °C to remove somatic cells. The supernatant was then centrifuged at 2000× *g* for 20 min at 4 °C to eliminate cellular debris. To remove fat, the supernatant was chilled at 4 °C for 30 min and centrifuged at 1500× *g* for 15 min, and the fat layer was discarded. The resulting supernatant was ultracentrifuged at 10,000× *g* for 30 min at 4 °C (Optima XE-90 Ultracentrifuge, Beckman Coulter, Type 70.1 Rotor, Brea, CA, USA) to pellet larger microvesicles. The supernatant was subsequently ultracentrifuged at 100,000× *g* for 70 min at 4 °C to pellet the EV fraction. The pellet was washed once with phosphate-buffered saline (PBS) and re-ultracentrifuged under the same conditions. Purified CM-EVs were resuspended in 100 μL PBS and stored at −80 °C until use. Protein concentration was determined using a bicinchoninic acid (BCA) assay kit (Pierce BCA Protein Assay Kit, Catalog No. 23225, Thermo Fisher Scientific, Waltham, MA, USA).

### 2.3. Characterization of CM-EVs

Nanoparticle tracking analysis (NTA): CM-EVs diluted 1:1000 in PBS were analyzed using a ZetaView PMX 110 system (Particle Metrix, Meerbusch, Germany) at 25 °C with a 90° detection angle. Each sample was measured three times to obtain mean hydrodynamic diameter and particle concentration.

Transmission electron microscopy (TEM): CM-EVs were fixed in 2% glutaraldehyde for 1 h at 4 °C, adsorbed onto Formvar-carbon coated copper grids (Electron Microscopy Sciences, Hatfield, PA, USA) for 10 min, and negatively stained with 2% uranyl acetate for 1 min. Images were captured using a Hitachi HT7800 microscope (Hitachi, Tokyo, Japan) operated at 80 kV.

Western blot: EV lysates (20 μg protein per lane) were separated by 10% SDS-PAGE and transferred to PVDF membranes (Bio-Rad, Catalog No. 1620177, Hercules, CA, USA). After blocking with 5% non-fat milk in TBST (50 mM Tris-HCl, 150 mM NaCl, 0.1% Tween-20, pH 7.6) for 1 h at room temperature, membranes were incubated overnight at 4 °C with primary antibodies against CD63 (1:1000), CD81 (1:1000), and Alix (1:1000). After washing, membranes were incubated with HRP-conjugated secondary antibodies (Catalog No. 7074, Cell Signaling Technology, Danvers, MA, USA) at a dilution of 1:5000 for 1 h at room temperature. Bands were visualized using enhanced chemiluminescence (ECL) substrate (Bio-Rad, Catalog No. 1705061, Hercules, CA, USA) on a ChemiDoc MP Imaging System (Bio-Rad, Hercules, CA, USA).

SDS-PAGE and Coomassie staining: EV lysates (20 μg) were resolved on 10% polyacrylamide gels and stained with Coomassie Brilliant Blue R-250 (Bio-Rad, Catalog No. 1610436, Hercules, CA, USA). Molecular weight markers (Bio-Rad, Catalog No. 1610374, Hercules, CA, USA) were used as standards.

### 2.4. Hydrogel Synthesis and EV Loading (System Optimization)

Chitosan (2% *w*/*v*, medium molecular weight, 50 kDa, deacetylation degree ≥ 75%, product No. C3646, Sigma-Aldrich, St. Louis, MO, USA) was dissolved in 0.1 M acetic acid (pH 5.0, analytical grade, Macklin Biochemical, Shanghai, China) under continuous stirring at 25 °C for 24 h, then cooled to 4 °C. Poloxamer 407 (10% *w*/*v*, product No. P2443, Sigma-Aldrich, St. Louis, MO, USA) was dissolved in deionized water at 50 °C with stirring and subsequently cooled to 4 °C. The cooled Poloxamer 407 solution was added dropwise to the cooled chitosan solution at 4 °C under gentle stirring. The pH of the mixture was adjusted to 7.0–7.4 using 0.1 M NaOH (analytical grade, Macklin Biochemical, Shanghai, China). The homogeneous precursor solution was stored at 4 °C. For EV-loaded hydrogel (CM-EVs–Gel), isolated CM-EVs (1 × 10^11^ particles/mL, as determined by NTA) were mixed with the hydrogel precursor at a 1:5 volume ratio (EV suspension to precursor) at 4 °C prior to gelation.

### 2.5. Hydrogel Characterization

Gelation time: The tube inversion method was used. One milliliter of sol was placed in a glass vial (Shanghai Titan Scientific Co., Ltd., Shanghai, China) and incubated in a 37 °C water bath (DK-8D, Shanghai Jinghong Experimental Equipment Co., Ltd., Shanghai, China). The gelation time was recorded as the time at which no flow was observed upon inversion (*n* = 3).

Injectability: CM-EVs–Gel sol (500 μL) was loaded into a 1 mL syringe (BD Biosciences, Franklin Lakes, NJ, USA) fitted with a 25-gauge needle (BD Biosciences, Franklin Lakes, NJ, USA) and extruded at a constant rate of 1 mL/min using a syringe pump (PHD 2000, Harvard Apparatus, Holliston, MA, USA). Successful extrusion without fracturing was recorded.

Stability: Pre-formed gel (500 μL) was placed in a 24-well plate (Corning Incorporated, Corning, NY, USA) and maintained at 25 °C for 48 h. Gel integrity was visually inspected, and weight loss was measured gravimetrically using an analytical balance (ME204, Mettler Toledo, Columbus, OH, USA) (*n* = 3).

### 2.6. In Vitro Release Kinetics

In vitro release of EVs from CM-EVs–Gel was evaluated using a dialysis bag method. CM-EVs–Gel (500 μL, containing 1 × 10^11^ particles/mL) was placed in a dialysis bag (100 kDa molecular weight cutoff, Spectrum Laboratories, Catalog No. 132720, Rancho Dominguez, CA, USA) and immersed in 10 mL PBS (pH 7.4, Gibco, Catalog No. 10010023, Thermo Fisher Scientific, Waltham, MA, USA) at 37 °C with gentle agitation (100 rpm) using an orbital shaker (TS-100, Grant Instruments, Cambridge, UK). At predetermined time points (1, 2, 4, 8, 12, 24, 36, 48 h), 200 μL aliquots of the release medium were withdrawn and replaced with an equal volume of fresh PBS. The concentration of EVs in the release medium was quantified by BCA protein assay (Pierce BCA Protein Assay Kit, Catalog No. 23225, Thermo Fisher Scientific, Waltham, MA, USA) and normalized to the initial loading. Cumulative release was expressed as the percentage of total encapsulated EVs. All experiments were performed in triplicate (*n* = 3). Release data were fitted to zero-order, first-order, Higuchi, and Korsmeyer-Peppas models using GraphPad Prism (version 9.0.0, GraphPad Software, San Diego, CA, USA) to identify the release mechanism.

### 2.7. In Vivo Safety and Transdermal Penetration

Acute ocular irritation: The test was conducted in New Zealand white rabbits (*n* = 6, female, 2.5–3.0 kg) following OECD Guideline 405. Approximately 100 mg of CM-EVs–Gel was placed into the conjunctival sac of one eye; the contralateral eye received no treatment and served as a control. Ocular responses (corneal opacity, iris inflammation, conjunctival redness and edema) were evaluated at 1, 24, 48, and 72 h using the Draize scoring system by blinded observers.

Chronic dermal toxicity: CM-EVs–Gel (500 mg per application) was applied daily to shaved dorsal skin of rabbits (*n* = 6) for 28 consecutive days. Control animals received blank hydrogel (same volume). Body weight, food consumption, and general behavior were monitored daily. At the end of the study, skin tissues from the application site were collected, fixed in 4% paraformaldehyde (PFA, Biosharp, Hefei, China), and processed for histopathological examination with hematoxylin and eosin (H&E) staining (Hematoxylin, Biosharp, Hefei, China; Eosin, Biosharp, Hefei, China).

Transdermal penetration: CM-EVs were labeled with PKH67 green fluorescent dye (PKH67 Green Fluorescent Cell Linker Kit, MINI67, Sigma-Aldrich, St. Louis, MO, USA) according to the manufacturer’s instructions. Briefly, 1 × 10^11^ CM-EVs were mixed with 1 μL of PKH67 dye in 250 μL of Diluent C (provided in the kit) for 5 min at room temperature. The reaction was stopped by adding 500 μL of 1% bovine serum albumin (BSA, Sigma-Aldrich, St. Louis, MO, USA), and unbound dye was removed by ultracentrifugation at 100,000× *g* for 70 min at 4 °C (Optima XE-90 Ultracentrifuge, Beckman Coulter, Type 70.1 Rotor, Brea, CA, USA). PKH67-labeled CM-EVs were incorporated into the hydrogel as described in Section 2.4 and applied to shaved dorsal skin of Sprague-Dawley rats (*n* = 6). Fluorescence imaging was performed at 1, 2, 4, and 8 h post-application using an IVIS Lumina III system (PerkinElmer, Waltham, MA, USA). Cryosections (10 μm) of treated skin were counterstained with DAPI (Vector Laboratories, Catalog No. H-1200, Newark, CA, USA), and fluorescence intensity in the epidermis and dermis was quantified using ImageJ (version 1.54f, National Institutes of Health, Bethesda, MD, USA).

### 2.8. Wound Healing Model and Efficacy Evaluation

**Animals:** Eight-week-old, specific pathogen-free Sprague-Dawley rats (220–250 g, both sexes) were sourced from Vital River Laboratory Animal Technology Co., Ltd. (Beijing, China). They were kept in standard conditions (12 h light/dark cycle, 22 ± 1 °C, 50 ± 10% humidity) with unlimited food and water. The Institutional Animal Care and Use Committee of Qinghai University School of Medicine approved all procedures (Protocol No. PJ202501-55, approved on 15 January 2025), adhering to the NIH Guide for the Care and Use of Laboratory Animals (8th edition, 2011).

Surgical procedure: Rats were anesthetized with pentobarbital sodium (50 mg/kg, intraperitoneally, Merck KGaA, Darmstadt, Germany) and placed on a 37 °C heating pad (Taimeng Instruments Co., Ltd., Chengdu, China). After shaving and cleaning the dorsal fur with depilatory cream (Veet, Reckitt Benckiser, Slough, UK) and 70% ethanol (analytical grade, Macklin Biochemical, Shanghai, China), two 6 mm full-thickness circular wounds were made on the dorsum using a sterile biopsy punch (Kai Industries Co., Ltd., Gifu, Japan). Hemostasis was maintained with sterile gauze (Winner Medical Co., Ltd., Shenzhen, China). In this study, animals were randomly allocated into three groups, each consisting of six subjects (*n* = 6 per group; comprising three males and three females per group): (1) CM-EVs–Gel, (2) blank hydrogel, and (3) untreated control (saline, 0.9% NaCl, analytical grade, Macklin Biochemical, Shanghai, China). Each treatment, with a volume of 100 μL per wound, was administered daily. All wounds were covered with sterile gauze (Winner Medical Co., Ltd., Shenzhen, China) and secured using hypoallergenic tape (3M Healthcare, St. Paul, MN, USA). The animals were housed individually and observed daily for any signs of infection or distress. Randomization was conducted using a random number table, and the investigators responsible for wound measurements and histological analyses were blinded to the group assignments. For wound closure assessment, wounds were photographed on days 0, 3, 6, 9, 12, and 15, alongside a reference scale (ruler, Deli Group Co., Ltd., Ningbo, China). Wound areas were traced and quantified using ImageJ (version 1.54f, National Institutes of Health, Bethesda, MD, USA). The percentage of wound closure was calculated using the formula [(A_0_ − A_t_)/A_0_] × 100, where A_0_ represents the initial wound area and A_t_ denotes the wound area at time t.

### 2.9. Histological and Immunofluorescence Analysis

On day 15, animals were euthanized by CO_2_ inhalation followed by cervical dislocation. Wound tissues, including a 2 mm margin of surrounding healthy skin, were excised, fixed in 4% paraformaldehyde (PFA, Biosharp, Hefei, China) for 24 h at 4 °C, dehydrated through a graded ethanol series (analytical grade, Macklin Biochemical, Shanghai, China), and embedded in paraffin wax (Leica Biosystems, Nussloch, Germany). Sections (5 μm) were cut using a rotary microtome (Leica RM2255, Leica Biosystems, Nussloch, Germany).

H&E staining: Deparaffinized sections were stained with hematoxylin and eosin (H&E; Hematoxylin, Biosharp, Hefei, China; Eosin, Biosharp, Hefei, China) to evaluate re-epithelialization and epidermal thickness. Epidermal thickness was measured at three randomly selected fields per section (*n* = 3 sections per wound) using ImageJ (version 1.54f, National Institutes of Health, Bethesda, MD, USA).

Masson’s trichrome staining: Collagen deposition was assessed using a Masson’s trichrome staining kit (Sigma-Aldrich, Catalog No. HT15, St. Louis, MO, USA). Collagen-positive area (blue) was quantified as a percentage of the total dermal area using ImageJ (version 1.54f, National Institutes of Health, Bethesda, MD, USA).

Immunofluorescence: Sections were dewaxed, rehydrated, and subjected to antigen retrieval by heating in sodium citrate buffer (10 mM, pH 6.0, Biosharp, Hefei, China) for 20 min. After blocking with 5% goat serum (Solarbio Life Sciences, Beijing, China) in PBS (Gibco, Catalog No. 10010023, Thermo Fisher Scientific, Waltham, MA, USA) for 30 min at room temperature, sections were incubated overnight at 4 °C with primary antibodies against ZO-1 (1:200, Invitrogen, Catalog No. 617700, Thermo Fisher Scientific, Waltham, MA, USA) and occludin (1:200, Invitrogen, Catalog No. OC-3F10, Thermo Fisher Scientific, Waltham, MA, USA). After washing, sections were incubated with Alexa Fluor 488-conjugated secondary antibodies (1:500, Invitrogen, Catalog No. A11008, Thermo Fisher Scientific, Waltham, MA, USA) for 1 h at room temperature. Nuclei were counterstained with DAPI (Vector Laboratories, Catalog No. H-1200, Newark, CA, USA). Fluorescence images were captured using a Zeiss Axio Imager Z2 microscope (Carl Zeiss, Oberkochen, Germany). Fluorescence intensity was quantified using ImageJ (version 1.54f, National Institutes of Health, Bethesda, MD, USA) and presented as fold change relative to the untreated control group.

### 2.10. Statistical Analysis

All data are expressed as mean ± standard deviation (SD). Statistical analyses were performed using GraphPad Prism 9.0 (GraphPad Software, San Diego, CA, USA). Prior to statistical analysis, the normality of data distribution was assessed using the Shapiro–Wilk test (*p* > 0.05 indicating normal distribution). Homogeneity of variances among groups was evaluated using Levene’s test (*p* > 0.05 indicating equal variances). For data that met both normality and homogeneity of variance assumptions, parametric tests were applied. Comparisons among three or more groups were conducted using one-way analysis of variance (ANOVA) followed by Tukey’s post hoc test for multiple comparisons. Two-group comparisons were performed using two-tailed Student’s *t*-test. For data that did not meet the assumptions of normality or equal variance, the corresponding non-parametric tests (Kruskal–Wallis test for multiple groups or Mann–Whitney U test for two groups) were used as appropriate. A value of *p* < 0.05 was considered statistically significant. All graphs include error bars (SD) and significance indicators (*p* < 0.05, *p* < 0.01, or *p* < 0.001) where applicable.

## 3. Results

### 3.1. Characterization of CM-EVs as Engineered Biogenic Particles

**Figure 1:**
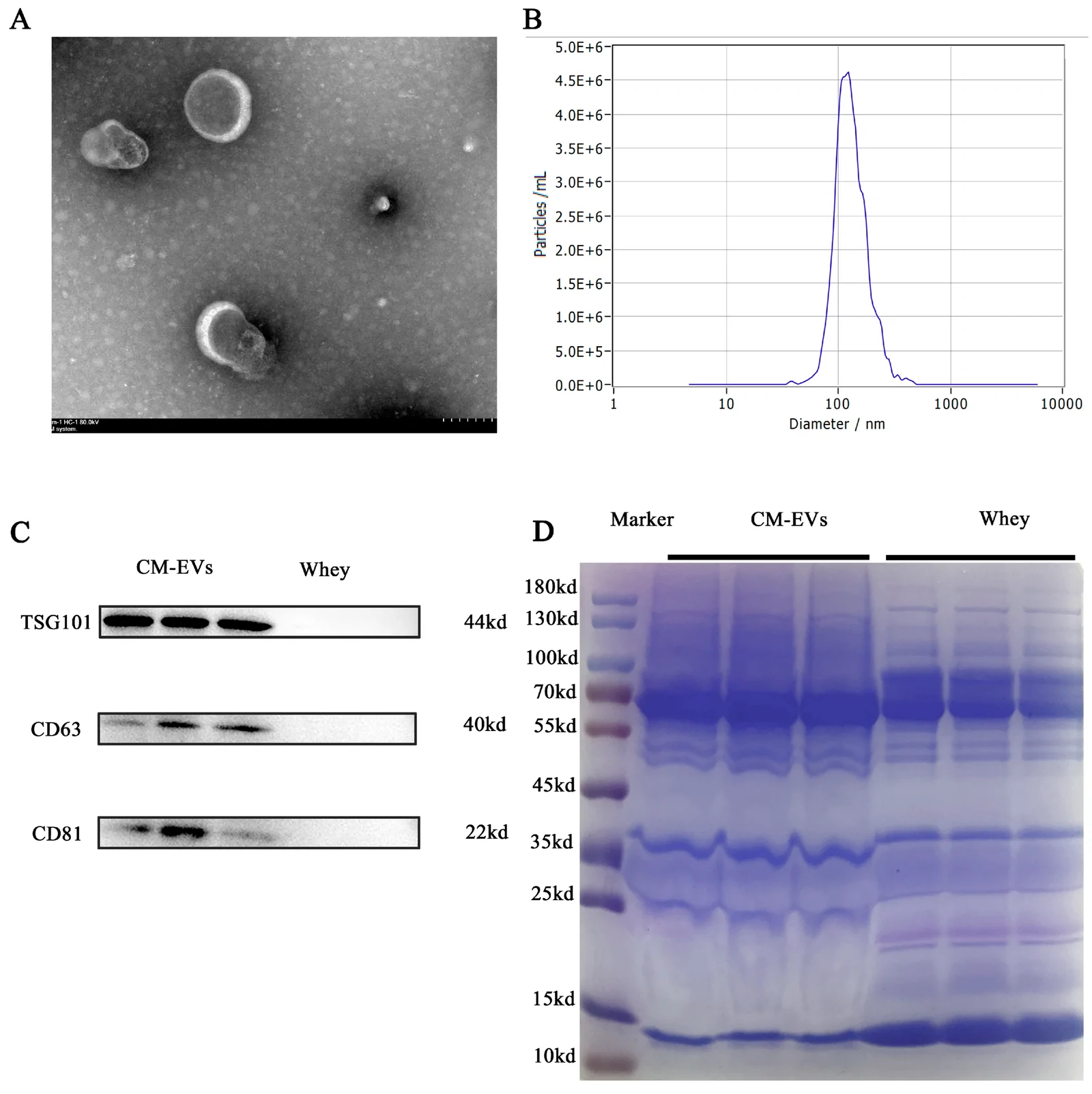
Characterization of CM-EVs. (**A**) Representative transmission electron microscopy (TEM) image showing the typical cup-shaped morphology of CM-EVs. Scale bar: 200 nm. (**B**) Nanoparticle tracking analysis (NTA) revealing the size distribution of CM-EVs, with a peak at 108.5 nm and a concentration of 1.11 × 10^11^ particles/mL. (**C**) Western blot analysis confirming the presence of the EV-associated markers CD63, CD81, and Alix in CM-EVs. (**D**) Coomassie blue-stained gel showing the protein profile of CM-EVs, with molecular weights ranging from 10 to 180 kDa.

Transmission electron microscopy (TEM) revealed typical cup-shaped morphology of CM-EVs, with diameters ranging from 30 to 150 nm (Figure 1A). Nanoparticle tracking analysis (NTA) demonstrated a monodisperse size distribution with a mean hydrodynamic diameter of 108.5 ± 12.3 nm and a particle concentration of (1.11 ± 0.08) × 10^11^ particles/mL (Figure 1B). Western blot analysis confirmed the expression of the exosomal surface markers CD63, CD81, and Alix (Figure 1C). SDS-PAGE with Coomassie staining revealed prominent protein bands at approximately 80 kDa (lactoferrin) and 25 kDa (TGF-β), along with additional bands at 70 kDa (HSP70) and 90 kDa (HSP90), confirming the presence of regenerative cargo within the engineered EV particles (Figure 1D).

### 3.2. Properties of the Optimized Thermosensitive Hydrogel System

**Figure 2:**
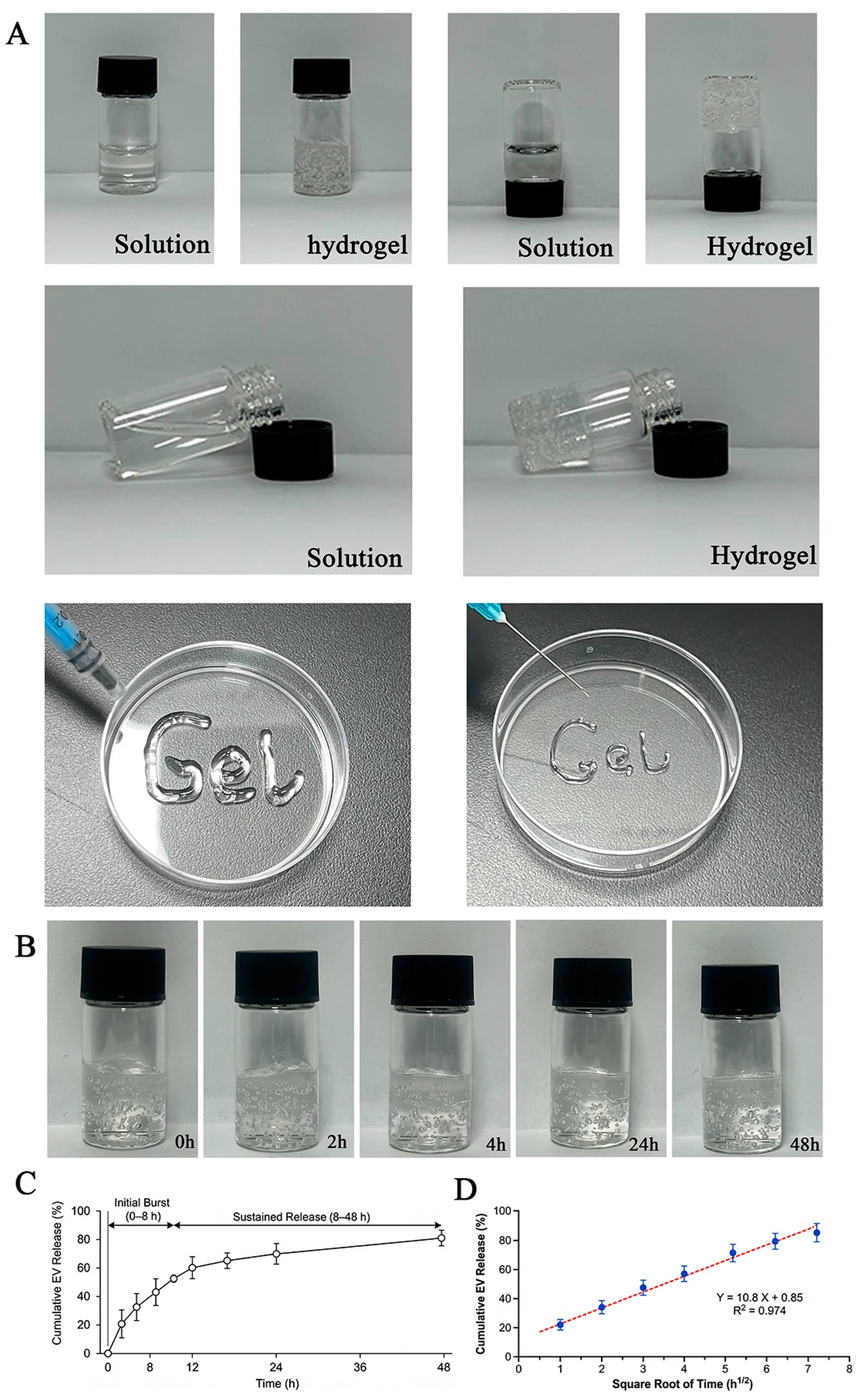
Properties and in vitro release kinetics of CM-EVs–Gel. (**A**) Extrusion of the gel through a 25-gauge needle without fracturing. (**B**) Weight loss (<8%) of the pre-formed gel maintained at 25 °C for >48 h. (**C**) Cumulative EV release profile over 48 h (43.2 ± 3.1% at 8 h, 81.7 ± 4.2% at 48 h). (**D**) Higuchi model fitting of release data ($R^{2}=0.974$), confirming diffusion-controlled release.

The CM-EVs–Gel formulation (2% chitosan/10% Poloxamer 407) exhibited rapid gelation at 37 °C, with a gelation time of 9.8 ± 0.5 min (*n* = 3) as determined by the tube inversion method. The hydrogel was easily injectable through a 25-gauge needle without fracturing (Figure 2A). Stability testing showed that the pre-formed gel maintained structural integrity at 25 °C for 48 h, with a cumulative weight loss of 7.6 ± 0.9% (*n* = 3) (Figure 2B), confirming system stability for practical handling and potential clinical application.

### 3.3. In Vitro Release Kinetics of the Integrated Particle–Hydrogel System

The cumulative release profile of EVs from CM-EVs–Gel over 48 h followed a biphasic pattern: An initial burst release of 43.2 ± 3.1% at 8 h, followed by a sustained release phase reaching 81.7 ± 4.2% at 48 h (*n* = 3) (Figure 2C). Release kinetic data were fitted to four mathematical models. The Higuchi model yielded the highest coefficient of determination ($R^{2}$ = 0.974), followed by the Korsmeyer–Peppas model ($R^{2}$ = 0.969), first-order model ($R^{2}$ = 0.941), and zero-order model ($R^{2}$ = 0.892) (Figure 2D). The best fit to the Higuchi model indicates a diffusion-controlled release mechanism characteristic of engineered particle–hydrogel composite systems.

### 3.4. System Safety and Transdermal Penetration

**Figure 3:**
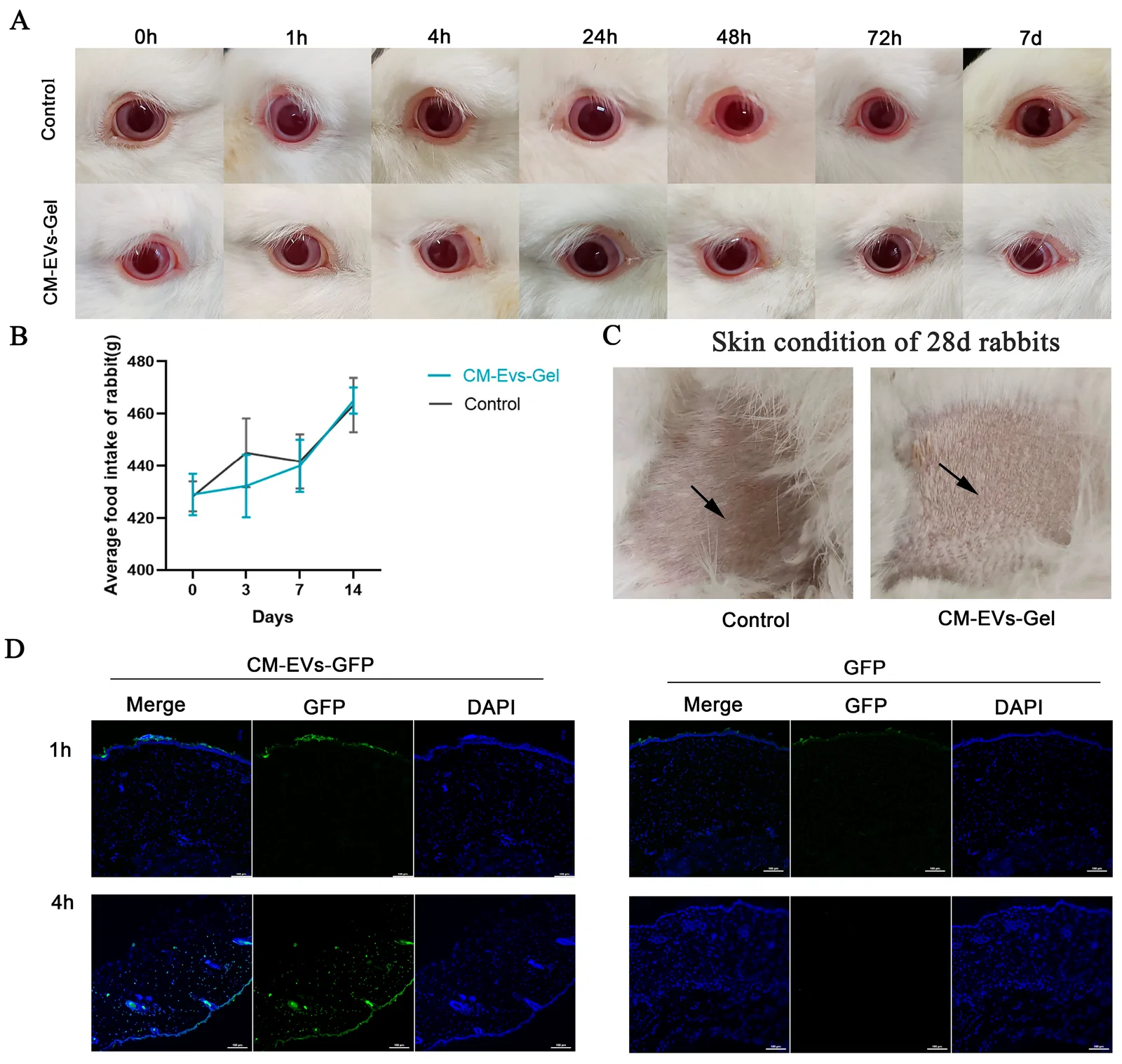
Biosafety and transdermal delivery of CM-EVs–Gel. (**A**) Acute ocular irritation test. (**B**) Feeding behavior during test. (**C**) 28-day chronic toxicity: normal skin histology, feeding, activity. Arrows indicate the representative observation areas. (**D**) Transdermal delivery: epidermal accumulation at 1 h, dermal penetration at 4 h (1 × 10^11^ particles/mL).

In the acute ocular irritation test (OECD Guideline 405), no corneal opacity, iris inflammation, conjunctival redness, or edema was observed at any time point (1, 24, 48, and 72 h). Draize scores were consistently 0/110 for all treated eyes (*n* = 6), confirming the non-irritant nature of CM-EVs–Gel (Figure 3A). In the 28-day chronic dermal toxicity study, no significant differences in body weight gain, food consumption, or general behavior were observed between CM-EVs–Gel-treated rabbits and blank hydrogel controls (*n* = 6 per group) (Figure 3B,C). Histological examination of treated skin sites revealed normal epidermal and dermal architecture with no signs of inflammation, necrosis, or fibrosis.

For transdermal penetration, PKH67-labeled CM-EVs incorporated into the hydrogel showed detectable green fluorescence in the epidermis as early as 1 h post-application. By 4 h, fluorescence intensity increased and was clearly visible in the dermis (Figure 3D). These results demonstrate that the thermosensitive hydrogel facilitates effective transdermal delivery of engineered EV particles.

### 3.5. Wound Healing Efficacy of the Complementary Particle–Hydrogel Strategy

**Figure 4:**
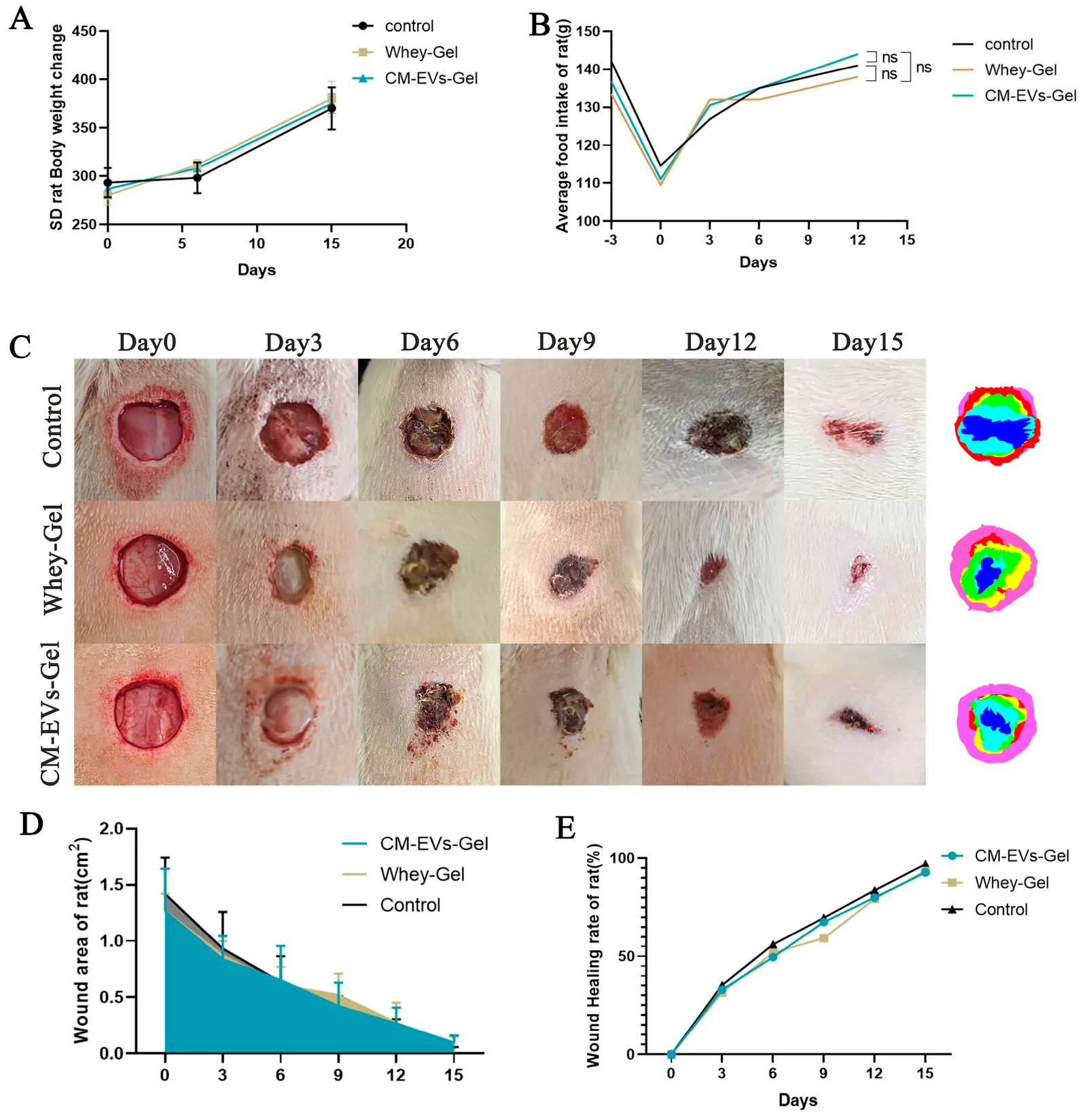
Wound healing efficacy of CM-EVs–Gel in rat models. (**A**) Body weight changes in SD rats during the treatment period. (**B**) Feeding behavior of SD rats during the treatment period; ns, not significant. (**C**) Representative images of skin wound healing at indicated time points. (**D**) Quantification of wound area over time. (**E**) Wound healing rate (percentage of initial wound area closure).

All rats completed the 15-day experiment without adverse events or signs of infection. No significant differences in body weight change or food intake were observed among the three groups (*p* > 0.05) (Figure 4A,B). Wound closure rates are summarized in Figure 4D,E. On day 6, the CM-EVs–Gel group achieved a wound closure rate of 76.31 ± 4.12%, which was significantly higher than the blank hydrogel group (48.77 ± 5.23%) and the untreated control group (43.14 ± 5.91%) (*p* < 0.01 for both comparisons). On day 15, closure rates reached 94.7 ± 2.8% for CM-EVs–Gel, 82.1 ± 4.5% for blank hydrogel, and 72.3 ± 6.1% for untreated controls (*p* < 0.01 for CM-EVs–Gel vs. blank hydrogel; *p* < 0.001 for CM-EVs–Gel vs. untreated control). Representative wound images at each time point are shown in Figure 4C (corrected images).

**Figure 5:**
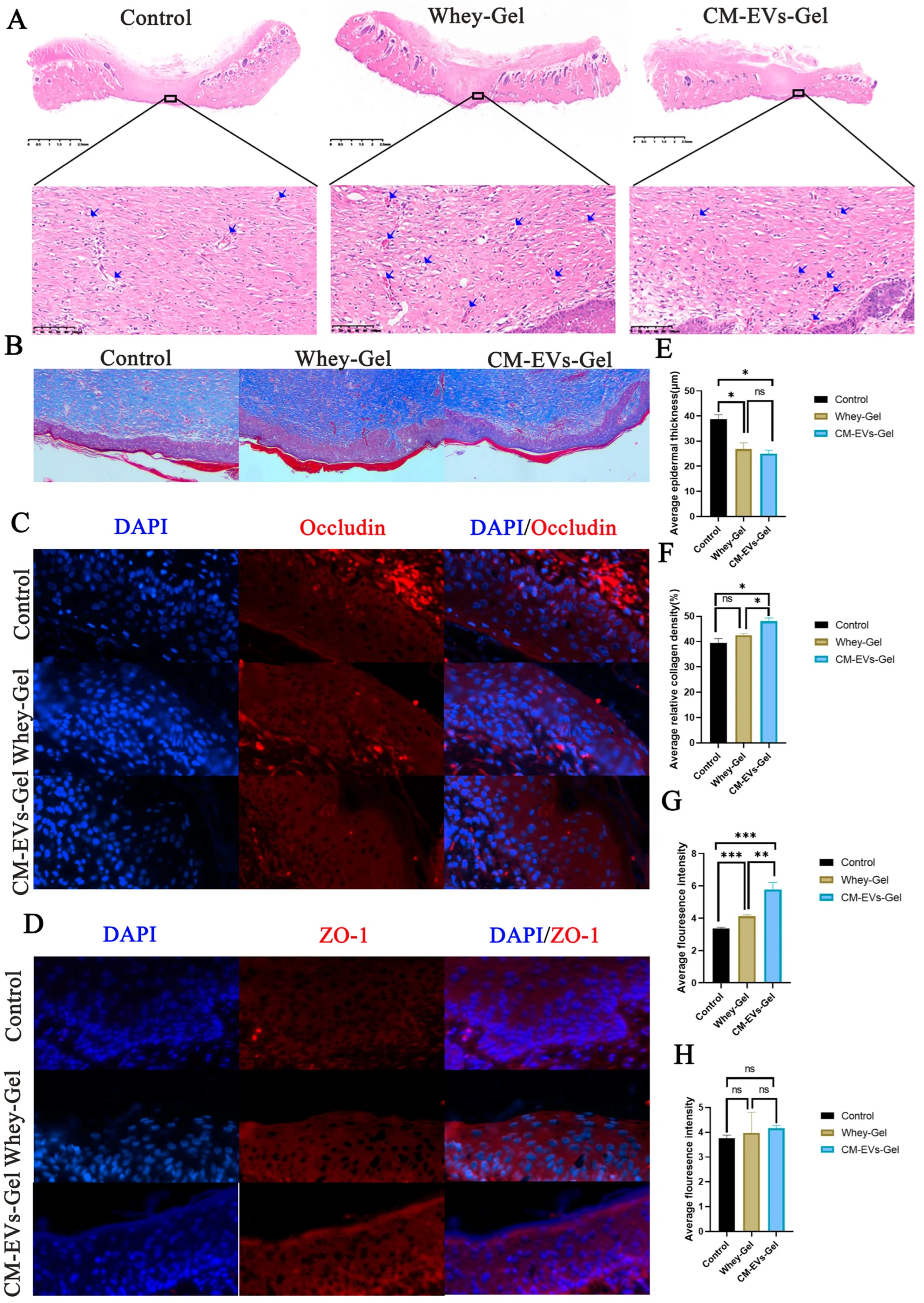
Wound healing efficacy of CM-EVs–Gel. (**A**) Representative hematoxylin and eosin (H&E) staining of wound tissues. Scale bar: 200 µm. (**B**) Representative Masson’s trichrome staining showing collagen deposition. Scale bar: 200 µm. (**C**,**D**) Immunofluorescence staining of tight junction proteins Occludin ((**C**), red) and ZO-1 ((**D**), red) in the wound epithelium. Nuclei were counterstained with DAPI (blue). Scale bars: 200 µm. (**E**) Quantitative analysis of epidermal thickness based on H&E staining. *p* < 0.05 vs. control group. (**F**) Quantitative analysis of collagen deposition based on Masson’s trichrome staining. *p* < 0.05 vs. control group. (**G**,**H**) Quantification of Occludin (**G**) and ZO-1 (**H**) fluorescence intensity, confirming enhanced expression of tight junction proteins. *p* < 0.05 vs. control group. * *p* < 0.05, ** *p* < 0.01, and *** *p* < 0.001 indicate statistically significant differences compared with the control group; ns indicates no significant difference (*p* ≥ 0.05).

Histological analysis of day-15 wound tissues (Figure 5) revealed that CM-EVs–Gel treatment resulted in complete re-epithelialization with a well-organized stratified epidermis, resembling normal skin architecture (Figure 5A). Epidermal thickness in the CM-EVs–Gel group was 28.4 ± 4.2 μm, representing a 1.8-fold increase compared to untreated controls (15.6 ± 3.1 μm, *p* < 0.01) (Figure 5E). Masson’s trichrome staining showed dense, regularly arranged collagen fibers in CM-EVs–Gel-treated wounds, with collagen deposition measured at 2.1 ± 0.3-fold higher than untreated controls (*p* < 0.01) (Figure 5B,F). New blood vessel density was 42.3 ± 6.8 vessels per high-power field in the CM-EVs–Gel group, which was 2.4-fold greater than in untreated controls (17.6 ± 4.2, *p* < 0.01). Immunofluorescence staining demonstrated pronounced expression of ZO-1 and occludin at the wound epithelial edge in the CM-EVs–Gel group (Figure 5C,D). Quantification revealed a 1.9 ± 0.3-fold increase in ZO-1 (*p* < 0.01) and a 1.2 ± 0.2-fold increase in occludin (*p* < 0.05) compared to untreated controls (Figure 5G,H).

## 4. Discussion

In the present study, we developed a thermosensitive chitosan/Poloxamer 407 hydrogel loaded with camel milk-derived extracellular vesicles (CM-EVs–Gel) and evaluated its wound-healing potential. Our results demonstrate that CM-EVs–Gel significantly accelerates wound closure in a rat full-thickness skin defect model, concomitant with enhanced re-epithelialization, collagen deposition, angiogenesis, and tight junction protein expression.

CM-EVs were successfully isolated by differential ultracentrifugation, yielding particles with a mean diameter of 108.5 nm, consistent with the typical size range of small EVs. The expression of canonical EV markers CD63, CD81, and Alix fulfilled the minimal characterization criteria recommended by MISEV2023 [23]. Nevertheless, differential ultracentrifugation—despite its widespread use and practical convenience—inherently affords limited purity, particularly when applied to complex biological fluids such as milk, wherein co-isolation of casein micelles, whey proteins, and lipoproteins remains a concern [24]. SDS-PAGE analysis revealed bands consistent with the molecular weights of several bioactive proteins, including lactoferrin, TGF-β, HSP70, and HSP90, within the CM-EV cargo. These proteins are functionally relevant to wound repair: lactoferrin exhibits antimicrobial and immunomodulatory properties [25]; TGF-β facilitates fibroblast activation and extracellular matrix synthesis [26]; and HSP70/90 exert paracrine effects that enhance cell survival and tissue regeneration [27]. However, protein identification based solely on electrophoretic mobility is provisional and warrants cautious interpretation, as it does not constitute definitive proof of the presence of these specific species. Our characterization strategy followed the MISEV-endorsed orthogonal approach, combining TEM, NTA, and Western blotting for EV identification. We did not, however, perform targeted Western blotting, ELISA, or mass spectrometry for lactoferrin, TGF-β, or other individual bioactive proteins, nor did we assess negative markers such as calnexin, GM130, or ApoA1 to exclude non-EV contaminants [24]. While additional purification techniques—density gradient centrifugation or size-exclusion chromatography—could enhance purity, they may compromise yield. We therefore recommend that future studies incorporate negative marker analysis, orthogonal purification strategies, and targeted proteomic assays (e.g., Western blotting with specific antibodies, ELISA, or LC-MS/MS) to definitively confirm the presence and abundance of lactoferrin, TGF-β, and other bioactive proteins in CM-EVs, particularly given the protein-rich and complex nature of milk as a starting material. Notwithstanding these limitations, our findings establish camel milk as a scalable and abundant natural source of EVs with considerable therapeutic promise for wound healing.

Topical application of free EVs is constrained by rapid proteolytic degradation, mechanical removal, and poor penetration across the stratum corneum [9]. The thermosensitive chitosan/Poloxamer 407 hydrogel developed herein addresses these obstacles through multiple mechanisms. The system undergoes a sol-to-gel transition at 37 °C within 10 min, enabling minimally invasive liquid administration followed by rapid in situ gelation. The resultant occlusive, semi-solid depot conforms to irregular wound beds and provides a physical barrier against exudate drainage and external contamination. Although we did not perform rheological measurements (a limitation acknowledged below), the gelation time and injectability data support its practical utility. Importantly, the chitosan component may confer additional protection to EVs through electrostatic shielding [27], potentially extending EV stability beyond that achievable with Poloxamer 407 alone [28].

The chitosan used in this study (C3646, Sigma-Aldrich) is a commercially available polymer with a molecular weight of approximately 90–190 kDa and a degree of deacetylation of ~75% [29]. Poloxamer 407 (P2443, Sigma-Aldrich) is a well-characterized thermosensitive triblock copolymer (PEO_99_–PPO_67_–PEO_99_, Mw ~12,600 Da) known for its reversible sol–gel transition at physiological temperature [18]. The physicochemical and rheological properties of the chitosan/Poloxamer 407 composite hydrogel system have been extensively documented [30]. Injectability was assessed qualitatively by visual confirmation of smooth extrusion through a 22-gauge needle at room temperature, consistent with the minimally invasive application envisioned clinically. We acknowledge that this assessment is descriptive and lacks quantitative injection-force data. However, given that numerous prior studies have demonstrated that similar chitosan/Poloxamer 407-based formulations exhibit injection forces well within clinically acceptable ranges under standardized testing conditions [31,32], we considered this qualitative confirmation sufficient for the primary objectives of the present study—namely, biological efficacy and therapeutic outcomes—rather than engineering optimization of the hydrogel per se. We have explicitly recommended in the Discussion that future investigations incorporate quantitative injectability testing, such as force–displacement profiling using a universal testing machine equipped with a syringe fixture, to further substantiate the translational potential of this system.

Accordingly, the present study focused on evaluating the biological performance and therapeutic efficacy of the CM-EV-loaded formulation, while referencing previously reported physicochemical characterization data for this hydrogel system. In vitro release studies revealed a biphasic EV release profile from CM-EVs–Gel, characterized by an initial burst (43.2% at 8 h) followed by a sustained release phase (81.7% at 48 h). This behavior is indicative of diffusion-controlled release from a hydrogel matrix and is consistent with findings from previous EV–hydrogel systems [28,29]. Among the four kinetic models evaluated, the Higuchi model provided the best fit (*R*^2^ = 0.974), corroborating that EV release is predominantly diffusion-driven. The cumulative release of approximately 82% over 48 h suggests that the majority of encapsulated EVs are liberated within a clinically relevant therapeutic window, during which the hydrogel matrix continues to afford protection.

CM-EVs–Gel exhibited a favorable safety profile, with no ocular irritation in the Draize assay and no systemic or local toxicity in the 28-day dermal toxicity study. These findings are consistent with the established biocompatibility of chitosan and Poloxamer 407, both approved for pharmaceutical and medical device applications [19,20]. Furthermore, fluorescently labeled CM-EVs delivered via the hydrogel penetrated the epidermis within 1 h and reached the dermis by 4 h, indicating that the system effectively overcomes the stratum corneum barrier—a common challenge in topical nanoparticle therapy [17].

In the rat full-thickness wound model, CM-EVs–Gel significantly accelerated wound closure compared to both blank hydrogel and untreated controls. At day 6, the closure rate was 76.31%, reaching 94.7% by day 15. These improvements were accompanied by a 1.8-fold increase in epidermal thickness, a 2.1-fold increase in collagen deposition, and a 2.4-fold increase in new blood vessel density. Notably, the blank hydrogel also exerted some therapeutic effect (48.77% closure at day 6), likely attributable to its occlusive and moisture-retentive properties, which are known to create a favorable microenvironment for wound healing. The superior performance of CM-EVs–Gel over the blank hydrogel thus suggests an additional contribution from the CM-EV cargo beyond the supportive effects of the hydrogel matrix alone.

To investigate whether the observed therapeutic benefits could be ascribed to EV penetration into wound tissue, we performed a transdermal penetration assay using PKH67-labeled CM-EVs. Fluorescence signals detected in the deeper layers of wound tissue indicated that CM-EVs may traverse beyond the superficial wound surface, potentially delivering bioactive cargo to regenerating tissue. Nonetheless, we acknowledge a methodological limitation: the absence of a dye-only control group (i.e., PKH67 dye subjected to the same purification and experimental procedures without EVs) precludes definitive exclusion of the possibility that some fluorescence signals originated from free dye aggregates or dye-labeled non-EV contaminants, rather than from intact labeled EVs. We have therefore interpreted our PKH67 fluorescence data with due caution, employing them primarily as a qualitative indicator of potential EV penetration capability rather than as a definitive quantitative measure of EV distribution. It is noteworthy that the PKH67 labeling results are consistent with and corroborated by the observed in vivo therapeutic efficacy and histological improvements, providing indirect yet convergent evidence for EV penetration and bioactivity. We recommend that future research incorporate appropriate dye-only controls and, ideally, employ complementary labeling techniques—such as DiR or near-infrared dyes, or EV-specific markers—to more rigorously validate EV transdermal penetration and biodistribution.

We further acknowledge the absence of a treatment group receiving free CM-EVs in the current experimental design. Consequently, we are unable to conclusively differentiate the individual contributions of the hydrogel matrix, the intrinsic biological activity of the EVs, and their potential synergistic effects on the overall therapeutic outcome. The primary aim of this study was to validate the feasibility and efficacy of the integrated CM-EVs–Gel delivery platform as a comprehensive system, rather than to conduct a direct comparison between free and encapsulated EVs. Moreover, from a translational perspective, free EVs are likely to undergo rapid clearance and degradation in the proteolytic wound environment, rendering hydrogel encapsulation a more clinically relevant and practical approach for ensuring local retention and sustained bioactivity.

The observed regenerative effects can be attributed to multiple components of the CM-EVs–Gel system, although the precise molecular mechanisms remain to be fully elucidated. Lactoferrin, which is abundant in CM-EVs, is known to promote endothelial proliferation and tube formation [14]; TGF-β can induce VEGF expression in fibroblasts and endothelial cells [25]; and chitosan itself may contribute to a pro-angiogenic microenvironment by upregulating VEGF secretion [32,33,34,35,36]. Additionally, the upregulation of tight junction proteins ZO-1 (1.9-fold) and occludin (1.2-fold) suggests that CM-EVs–Gel not only supports structural wound closure but also facilitates functional restoration of the epidermal barrier—a finding consistent with reports that EVs can enhance tight junction assembly via miRNA and protein delivery [37]. These observations are congruent with the involvement of multiple regenerative pathways, including angiogenesis, extracellular matrix remodeling, and barrier function restoration.

We recognize that the present data are predominantly descriptive and histological, demonstrating enhanced re-epithelialization, collagen deposition, and angiogenesis, but they do not directly clarify the molecular pathways through which CM-EVs exert these effects. The observed upregulation of tight junction proteins and increased vascular density aligns with—but does not confirm—the involvement of specific signaling cascades. We have therefore interpreted our findings with due caution, refraining from definitive assertions regarding specific molecular mechanisms and presenting our results as a preliminary basis for future mechanistic studies. We recommend that subsequent research employ targeted methodologies—such as ELISA or multiplex assays for inflammatory cytokines (e.g., TNF-α, IL-6, IL-10) and oxidative stress markers, as well as Western blotting or immunohistochemistry for critical signaling pathway components (e.g., phosphorylated Akt, Smad2/3, ERK1/2)—to definitively elucidate the molecular mechanisms underlying CM-EV-mediated wound healing. We propose that in vitro investigations using relevant cell types (keratinocytes, fibroblasts, and endothelial cells), in conjunction with specific pathway inhibitors or siRNA knockdown, would be instrumental in systematically elucidating the causal roles of individual signaling pathways. Despite the limitations acknowledged, the robust and consistent efficacy data presented in this study establish a solid foundation for future mechanistic exploration of the CM-EVs–Gel system.

Previous research has employed mesenchymal stem cell-derived EVs embedded in thermosensitive hydrogels for wound healing applications [28]. Although these systems have similarly shown enhanced wound closure accompanied by increased angiogenesis and collagen deposition, their clinical application is limited by the high cost and complexity associated with stem cell culture [33]. Additionally, numerous prior studies have utilized an extensive array of immunohistochemical markers—such as CD31 for angiogenesis, Ki-67 for cell proliferation, and CD68/CD206 for macrophage polarization—to gain deeper mechanistic insights into regenerative processes. In contrast, our current study, while effectively demonstrating functional tissue regeneration through quantitative morphometric analyses—including assessments of epidermal thickness, collagen deposition, and blood vessel density—does not incorporate these specific immunohistochemical markers [34,35]. We recommend that future investigations integrate these markers to more thoroughly elucidate the cellular and molecular mechanisms underlying tissue regeneration mediated by CM-EVs. Despite this limitation, camel milk serves as an accessible, cost-effective, and scalable source of EVs, as a single camel can produce several liters of milk daily, facilitating large-scale EV production. Consequently, CM-EVs–Gel presents a potentially more economical and accessible alternative to stem cell-based EV systems, with the additional benefit of a sustainable and cost-efficient raw material supply chain.

**Figure 6:**
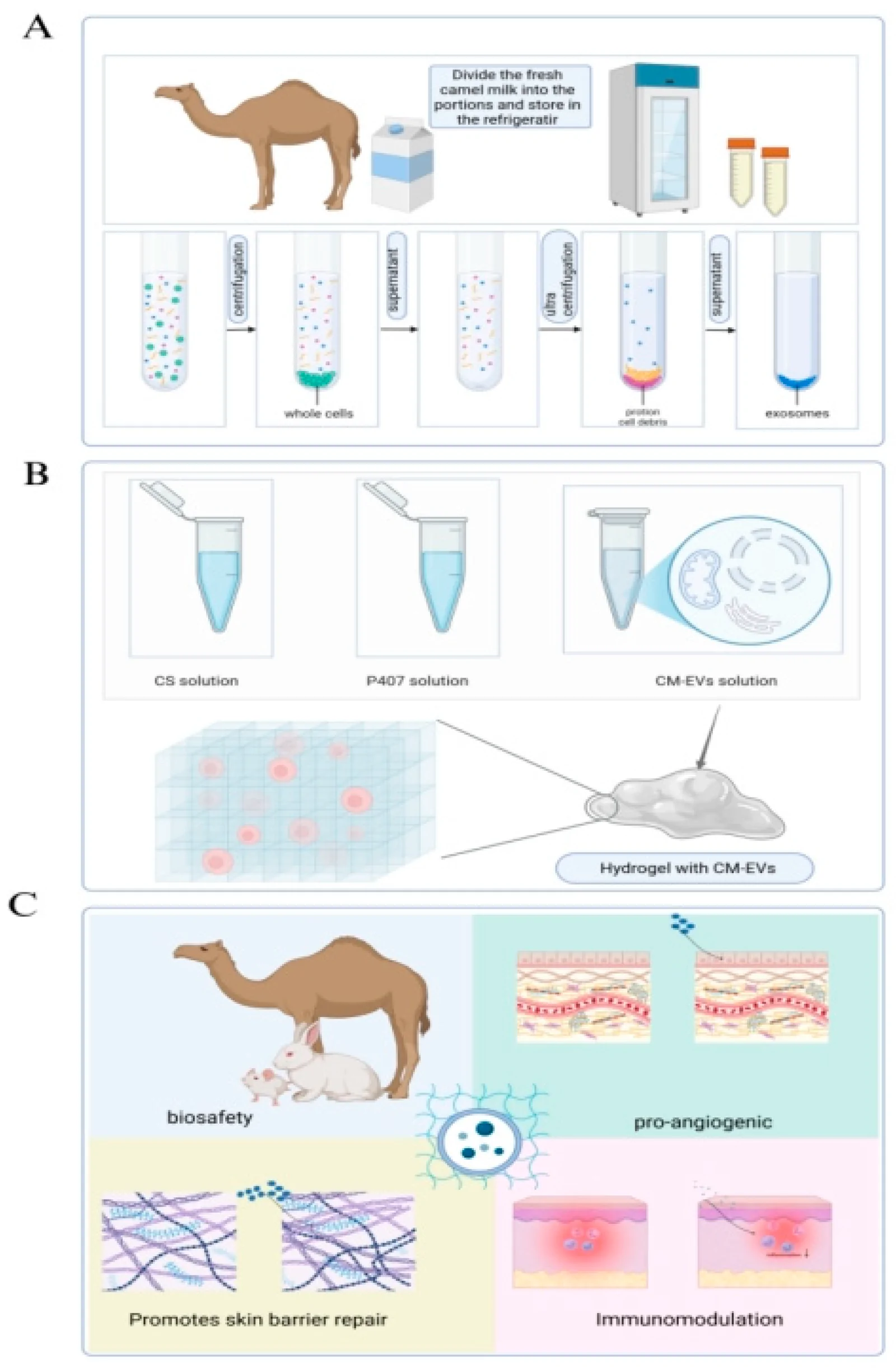
Schematic overview of CM-EVs preparation, hydrogel loading, and proposed mechanism in skin repair. (**A**) Preparation of CM-EVs by differential centrifugation (low-speed, high-speed) and cryopreservation. (**B**) Loading of CM-EVs into chitosan/Poloxamer 407 hydrogel to form CM-EVs–Gel. (**C**) Schematic of CM-EVs–Gel for skin repair, showing enhanced barrier function, blood supply, anti-inflammation, and biosafety.

Although preclinical results demonstrate that the CM-EVs–Gel system is safe and effective, several key challenges remain before clinical translation. Developing scalable, cost-effective, and GMP-compatible methods for CM-EV isolation is crucial, as current methods such as differential ultracentrifugation are limited in throughput and scalability. Additionally, the complex composition of camel milk—high in fat and protein—complicates purity achievement. Promising new strategies, including tangential flow filtration, PEG-based precipitation with chromatographic purification, and rennet-assisted casein precipitation, require further validation for camel milk applications. The inherent instability of EVs during storage further complicates clinical logistics; lyophilization has emerged as a promising strategy to enhance long-term stability. Recent studies have demonstrated that lyophilized camel milk exosomes preserved with trehalose, mannitol, and glycine retain their physicochemical properties and over 90% of protein content after 8 weeks of storage at −80 °C, suggesting a viable path toward an off-the-shelf product [38]. Batch-to-batch consistency poses another major challenge, given the intrinsic variability in camel milk due to factors such as animal breed, age, health status, lactation stage, and environmental conditions [39]; rigorous quality control parameters, including standardized isolation protocols, comprehensive characterization via orthogonal techniques (NTA, TEM, Western blot), and functional bioactivity assays, will be essential to ensure clinical reproducibility. Sterilization presents a further translational hurdle, as conventional terminal sterilization methods are likely to compromise EV integrity, making aseptic processing throughout the entire manufacturing chain the most viable approach, with sterile filtration through 0.22-μm filters as a potential alternative [40]. Finally, the regulatory pathway for CM-EV-based therapeutics remains incompletely defined; however, compliance with MISEV2023 guidelines, comprehensive characterization of EV identity and purity, rigorous toxicology and biodistribution studies, and proactive dialogue with regulatory authorities will be foundational for regulatory approval [40]. Despite these challenges, recent technological advances in scalable isolation, lyophilization, and membrane-based separation offer encouraging solutions, and continued interdisciplinary research at the interface of EV biology, biomaterials science, and pharmaceutical engineering will be essential to accelerate the clinical translation of this and related EV-based therapeutic platforms (Figure 6).

To address the aforementioned limitations and further advance this line of research, future studies should: (i) include a free EV control group and a dose–response evaluation; (ii) perform rheological measurements to fully characterize the thermosensitive behavior; (iii) quantify EV integrity and release kinetics in wound exudate or in vivo; (iv) conduct in vitro assays (scratch, proliferation, tube formation) to directly assess EV bioactivity; (v) investigate signaling pathways via Western blot or qPCR; (vi) evaluate systemic toxicity with comprehensive blood panels and organ histology; and (vii) test the system in diabetic animal models (e.g., db/db mice, STZ-induced diabetic rats) with inclusion of both sexes.

## 5. Conclusions

In conclusion, this study validates a complementary drug delivery strategy combining biogenic particle engineering (CM-EVs) and thermosensitive hydrogel system optimization for enhanced wound healing. Engineered CM-EV particles exhibit optimal size, morphology, and regenerative bioactivity, while integration into a thermosensitive hydrogel enables injectable, in situ gelling, sustained release, and improved skin delivery. In vivo results confirm safety and accelerated wound closure through enhanced re-epithelialization, collagen deposition, and angiogenesis. While preliminary, this work aligns closely with the special topic’s core focus on integrated delivery strategies spanning particle design to system-level performance, offering a scalable, nature-inspired platform for regenerative medicine and complementary drug delivery applications. Further mechanistic and translational studies are required to fully validate its clinical potential.

## Data Availability

The data presented in this study are available on request from the corresponding author due to privacy/ethical restrictions.

## Abbreviations

The following abbreviations are used in this manuscript:

ANOVA	Analysis of variance
BCA	Bicinchoninic acid
CM-EVs	Camel milk-derived extracellular vesicles
DAPI	4′,6-Diamidino-2-phenylindole
EV(s)	Extracellular vesicle(s)
H&E	Hematoxylin and eosin
HPF	High-power field
HSP	Heat shock protein
MISEV	Minimal Information for Studies of Extracellular Vesicles
MWCO	Molecular weight cut-off
NIH	National Institutes of Health
NTA	Nanoparticle tracking analysis
OECD	Organisation for Economic Co-operation and Development
PBS	Phosphate-buffered saline
PKH67	A green fluorescent cell linker dye
RIPA	Radioimmunoprecipitation assay
SD	Standard deviation
SDS-PAGE	Sodium dodecyl sulfate-polyacrylamide gel electrophoresis
TEM	Transmission electron microscopy
TGF-β	Transforming growth factor-beta
VEGF	Vascular endothelial growth factor
ZO-1	Zonula occludens-1
